# Rational Design of Hierarchical Beta Zeolites via Post-Synthesis Treatments and Their Applications

**DOI:** 10.3390/molecules30051030

**Published:** 2025-02-24

**Authors:** Michał Zieliński, Natalia Matysiak, Ewa Janiszewska

**Affiliations:** Faculty of Chemistry, Adam Mickiewicz University, Uniwersytetu Poznańskiego 8, 61-614 Poznań, Poland; mardok@amu.edu.pl (M.Z.); natmat6@st.amu.edu.pl (N.M.)

**Keywords:** hierarchical zeolite BEA, post-synthesis modification, NaOH, NH_4_OH, NH_4_F, esterification of acetic acid, methylene blue (MB) adsorption

## Abstract

Hierarchical zeolites with micro- and mesoporous frameworks can overcome diffusional limitations of microporous systems. This study investigates the post-synthetic modification of Beta zeolite using different porogeneous agents (NaOH, NH_4_OH, NH_4_F) under identical conditions to compare their efficiency in generating mesopores. The effect of treatment time was also examined for NH_4_OH and NH_4_F. The modified materials were characterized using physicochemical techniques and evaluated for catalytic performance in acetic acid esterification with alcohols of different sizes and adsorption of methylene blue. All the modifications increased mesoporosity but reduced acidity. NaOH produced the highest mesoporosity but significantly reduced acidity, while NH_4_F retained the most acidity. Catalytic activity in esterification with methanol depended on acidity, but for larger alcohols (n-butanol, benzyl alcohol), activity was influenced by both acidity and mesoporosity. The NH_4_OH- and NH_4_F-modified materials, with lower mesoporosity but higher acidity, exhibited better performance with larger alcohols. In MB adsorption, the adsorption equilibrium rates increased with mesoporosity. The NaOH-modified sample reached equilibrium the fastest due to its superior mesoporosity, while the NH_4_F-modified sample demonstrated the highest adsorption efficiency owing to its abundant Brønsted acid sites. These findings demonstrate that the choice of modifier affects mesoporosity, acidity, and functional performance, offering insights into tailoring hierarchical zeolites for specific applications.

## 1. Introduction

Zeolites, crystalline aluminosilicates with an ordered porous structure, play a key role in numerous chemical processes. Their high specific surface area, ion exchange properties, strong acidity, shape selectivity, and remarkable thermal and hydrothermal stability make them widely employed as catalysts in organic transformations such as cracking, isomerization, and esterification, as well as ion exchangers and adsorbents in purification and separation processes [1,2,3,4,5]. Their structural versatility, including tunable pore sizes and the potential for chemical modifications, makes them highly versatile materials in science and industry. However, their small pore dimensions impose diffusion limitations when the sizes of penetrating molecules are comparable to the rigid zeolite pore apertures. This limitation can restrict access to active sites within micropores, increase the likelihood of coking during catalytic reactions, and ultimately hinder their use in applications involving larger molecules [6].

To address these challenges, various strategies have been developed. One example involves the production of nanocrystalline zeolites, which enhances the external surface area and reduces diffusion path lengths [7,8,9]. Another method is the synthesis of extra-large pore zeolite with more than 12–member rings [10,11]. Recently, there has been great interest in developing microporous materials with additional porosity, often in the mesopore range, to enhance diffusion to internal active sites [12,13,14,15,16,17]. Hierarchically structured zeolites can be obtained through various approaches, including post-synthesis treatments (top-down methods) and direct synthesis of hierarchical zeolites (bottom-up methods) by using hard (e.g., carbon) or soft templates (e.g., surfactants) [12,18,19]. Among these strategies, post-synthesis treatments and the use of solid carbon templates have proven particularly effective in producing mesoporous zeolites. However, the carbon template method is not suitable for large-scale production due to its complex synthesis procedures and the hydrophobic nature of the templates.

The post-synthesis modification of zeolites, such as dealumination and desilication, offers simple, practical, and highly effective approaches to enhancing their properties. Dealumination, achieved through steam or acid treatment, introduces additional porosity by creating defect sites within the zeolite framework while simultaneously altering its acidic properties [20,21]. Desilication, performed using alkaline solutions, has been proved to be a more effective post-treatment method to improve the porosity of zeolites by selectively removing silicon atoms from the framework. However, this process is associated with certain drawbacks, including material loss, reduced crystallinity, decreased microporosity, and altered acidity [13,22]. These drawbacks can be minimized by replacing NaOH with tetraalkylammonium hydroxides (e.g., TPAOH or TBAOH) or by using mixtures of NaOH and alkylammonium hydroxides or surfactants [21,22,23,24]. However, the mesoporosity formed using these modified treatments is generally less pronounced than that achieved with pure NaOH treatment. Furthermore, the high cost and potential toxicity of these organic compounds reduce the feasibility of producing hierarchical zeolites on a large scale using these methods.

An alternative approach to addressing the limitations of desilication and dealumination is fluoride etching, which has shown significant promise. This method involves the post-synthetic treatment of zeolites using an aqueous solution of ammonium fluoride (NH_4_F) and/or hydrofluoric acid (HF) to create a hierarchical structure while preserving the zeolite framework and maintaining its chemical composition [25,26,27]. Under equilibrium conditions, the NH_4_F and/or HF aqueous solution provides various fluoride species, leading to similar rates for the dissolution of silica and alumina sites in the zeolite framework. This ensures that the crystallinity and acidity of the parent zeolites are preserved during the process [28,29].

Zeolite Beta (*BEA type) stands out within the diverse family of zeolites as one of the key materials used in the petrochemical and chemical industries. It exhibits catalytic activity in a wide range of reactions, including cracking, alkylation, isomerization, and aromatic acylation [30,31,32,33,34]. As one of the “Big Five” zeolites predominantly used in industrial applications [35], zeolite Beta possess large micropores (0.55 × 0.55 nm and 0.76 × 0.64 nm) with a three-dimensional 12-membered ring (12-MR). Its framework consists of randomly intergrown polymorphs, resulting in two straight channels and one oscillating channel, which enhance its versatility and catalytic performance. Beyond its excellent structural properties, Beta zeolite is a highly attractive catalyst due to its optimal balance of Brønsted and Lewis acid sites [36,37]. However, despite its relatively large pores, Beta zeolite often exhibits limited diffusion and restricted access to active sites when processing large reactant or product molecules. This limitation results in reduced catalytic efficiency and slower reaction rates, driving increasing interest in the development of hierarchical Beta zeolites. The strategy to obtain hierarchical Beta zeolites has explored several aspects, including the selection of template materials, tailoring mesopore sizes through base leaching, organotemplate-free synthesis, and selective desilication. Research on the synthesis of hierarchical Beta zeolites has primarily focused on templated strategies involving various organic hard and soft templates. In contrast, innovations in top-down synthesis have been limited due to the low framework stability of Beta zeolites in alkaline solutions [38,39]. In bottom-up methods, hierarchical Beta crystals have been synthesized using carbon-based materials such as carbon black, nanotubes, nanofibers, aerogels, and ordered mesoporous carbon replicas as hard templates [40,41,42,43]. Hard templates often result in materials with larger pores, typically in the macropore range, due to the size of the template molecules around which the zeolite forms. In contrast, the creation of hierarchical Beta nanocrystals with smaller mesopore sizes (2–5 nm) has been achieved using soft templates such as organosilanes or silylated polymers and the pore sizes reflect the dimensions of these soft template molecules [44,45,46]. Soft template synthesis can also employ cationic polymers [38,39,47]. In cases where the zeolite precursor gel is sufficiently concentrated, hierarchical Beta zeolite can be synthesized without the use of mesoporous templates through the aggregation of nanocrystals [48,49]. Top-down methods are more frequently applied in zeolite hierarchization due to their simplicity. However, because Beta zeolite is less stable in alkaline solutions [50,51], various strategies have been developed to overcome this challenge. These include traditional desilication and desilication with a pore-directing agent (PDA) [52,53]. Good results have been achieved with desilication assisted by pore-directing agents (PDAs). Research has shown that PDAs, such as tetrapropylammonium or cetyltrimethylammonium cations, exhibit protective properties toward Beta zeolite by reducing its surface exposure during desilication with NaOH. This protective effect is attributed to the preferential interaction between PDA molecules and the zeolite surface through hydrophobic forces. Moreover, it has been observed that the external surface area and average mesopore sizes of hierarchical Beta zeolites can be tailored by selecting PDAs of different sizes [54,55]. Given the simplicity and cost-effectiveness of conventional post-synthesis treatments and taking into account the destructive nature of base leaching, various base treatment conditions (e.g., the type of base such as NaOH, NH_4_OH, or PDAOH, as well as base concentration, modification time, and temperature) have been explored to produce hierarchical Beta zeolites with desirable textural and acidic properties. Studies have demonstrated that mesopore formation with preserved crystallinity requires alkaline conditions under atmospheric pressure, low temperatures (e.g., 65–80 °C), and short treatment durations (e.g., 30 min). However, many studies focus on a single type of mesopore-forming agent without comparing its performance to others. Additionally, variations in experimental setups and characterization conditions often make it difficult to directly compare the effectiveness of different agents. This inconsistency limits the broader understanding of optimal strategies for hierarchical Beta synthesis.

Considering the above, this study presents a comparative analysis of Beta zeolite hierarchization using different modifiers through post-synthesis modifications. The effects of the type of modifier solution and the treatment time on the textural properties and acidity of the resulting materials were evaluated. Given the lower stability of Beta zeolite in alkaline solutions compared to less open-structure zeolites, such as ZSM-5, ammonium hydroxide was selected as a weaker base alternative to the commonly used sodium hydroxide. Additionally, ammonium fluoride was chosen due to its reported ability to simultaneously remove silicon and aluminum, which preserves the original Si/Al ratio and, consequently, the acidity of the zeolite. For comparison, sodium hydroxide, the most commonly employed base for zeolite hierarchization, was also tested. The same modification conditions were used for all three modifiers, namely, 0.2 molar solutions at 65 °C for 0.5 h, reflecting the conditions most frequently reported in the literature for Beta zeolite modification. Given the well-preserved structure observed after modification with the NH_4_OH and NH_4_F solutions, longer treatment times (1 h and 2 h) were additionally investigated for these two modifiers. The resulting samples were characterized in terms of chemical composition, crystallinity, textural properties, and acidity. Their catalytic performance was assessed in the esterification of acetic acid with alcohols of varying molecular sizes, paying particular attention to the role of generated mesoporosity in facilitating the diffusion of reactants. Additionally, the materials’ adsorption capacity for methylene blue, used as a model bulky molecule in water purification processes, was evaluated to examine the impact of hierarchical porosity on adsorption efficiency.

## 2. Results and Discussion

**Figure 1:**
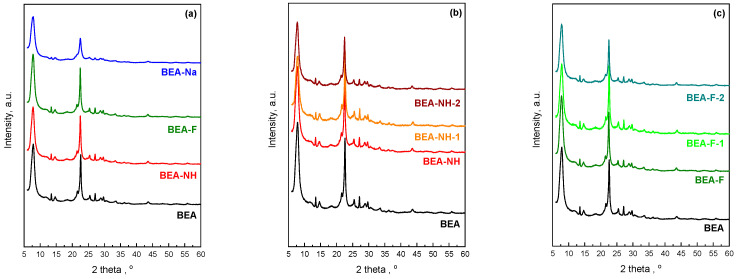
XRD patterns of zeolite Beta before and after modification: modified with different modifiers for 0.5 h (**a**), modified with NH_4_OH (**b**), and modified with NH_4_F (**c**) for different time.

The diffraction patterns of the parent and treated samples, shown in Figure 1, display characteristic intense 2θ peaks at 7.8° and 22.4°, typical of a Beta structure [56]. No significant changes were observed in the diffractograms following the modification of the starting material. However, some modified samples exhibited reflections of lower intensity, indicating a reduction in crystallinity relative to the parent zeolite during modification.

Table 1 presents the percentage crystallinity of the modified samples relative to the parent zeolite, which is considered 100% crystalline—BEA. Among the samples treated for 0.5 h, the sample modified with a NH_4_OH solution exhibits only a slight reduction in crystallinity compared to the parent sample. The most significant decrease in crystallinity is observed for the sample treated with a NaOH solution (BEA-Na), which exhibits a reduction of over 40%. In contrast, the sample modified with an NH_4_F solution for 0.5 h (BEA-F) shows a slight increase in crystallinity. This increase is attributed to the removal of low-crystallinity domains due to fluoride ion treatment, aligning with findings reported in Ref. [57]. However, extending the treatment time with NH_4_F solution results in a gradual decrease in crystallinity to 94% after 1 h and 92% after 2 h of modification. This reduction is less pronounced compared to the samples treated with the NH_4_OH solution, where crystallinity decreases more significantly, to 76% and 67% for 1 h and 2 h treatments, respectively.

**Figure 2:**
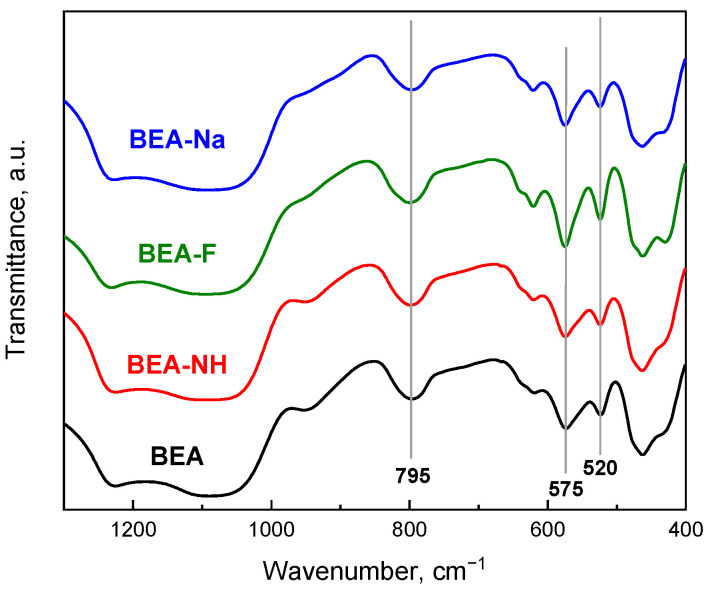
FTIR spectra of skeletal vibration of zeolite Beta before and after modifications.

The skeletal vibration FTIR spectra of the initial zeolite Beta and samples modified for 0.5 h with the NaOH, NH_4_OH, and NH_4_F solutions are shown in Figure 2. The spectra of the modified samples closely resemble the spectrum of the parent sample. The bands at 575 cm^−1^ and 525 cm^−1^, attributed to the vibrations of double rings characteristic of the zeolite Beta structure [50,58], confirm the correct BEA structure of the modified samples. However, a decrease in the intensity of these bands is observed for the BEA-NH and BEA-Na samples, indicating partial disruption of their structure. The band at ~800 cm^−1^ corresponds to the O–T–O symmetric stretching vibration (T = Si or Al), and its shift reflects changes in the framework Si/Al molar ratio [59], i.e., the wavenumber decreases with a decrease in the silicon content within the zeolite framework. For the initial sample, this band appears at 795 cm^−1^ and shifts slightly to 794 cm^−1^ for the modified samples, indicating only minor changes in the Si/Al ratio due to the extraction of framework silicon atoms during modification. Only the sample modified with NaOH exhibits a more significant shift to 791 cm^−1^, suggesting a higher degree of framework silicon extraction, consistent with the XRD results.

**Figure 3:**
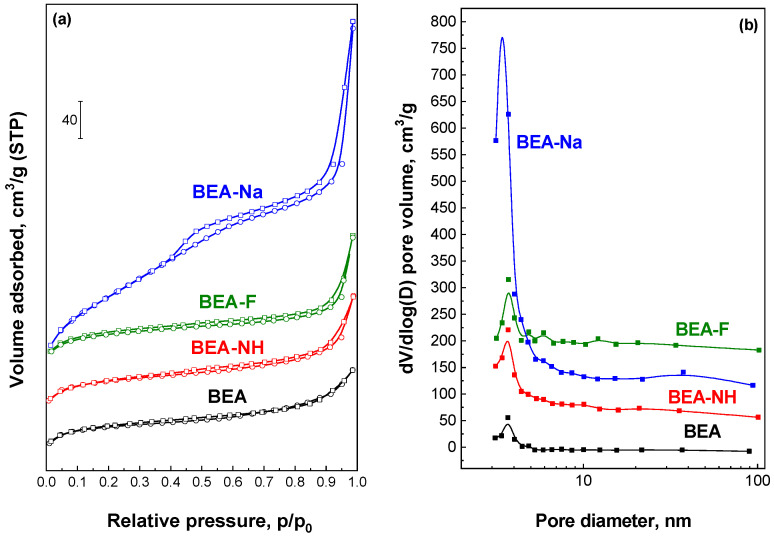
N_2_ adsorption/desorption isotherms (**a**) and pore size distribution (**b**) for initial and modified for 0.5 h BEA materials.

The textural properties of the initial and modified BEA samples were characterized using low-temperature nitrogen adsorption–desorption measurements. The results are summarized in Table 1, while the nitrogen adsorption–desorption isotherms and pore size distributions are presented in Figure 3, Figures S1 and S2.

The initial BEA sample exhibits a type I nitrogen adsorption—desorption isotherm (Figure 3a) with an uptake at low relative pressure (p/p_0_ < 0.1) and another increase at high relative pressure (p/p_0_ > 0.85). The uptake at low relative pressure indicates the microporous nature of the BEA sample, with a micropore volume of 0.23 cm^3^/g, typical of zeolites with a BEA structure [23]. The step observed in the p/p_0_ > 0.8 range can be attributed to the presence of interparticle voids resulting from the disordered agglomeration of BEA crystals. Such isotherms are characteristic of microporous zeolites with large crystals [60]. The small hysteresis loop in the p/p_0_ = 0.3–0.6 range, along with the relatively high external surface area (S_ext_ = 74 m^2^/g), further confirms the contribution of only interparticle voids formed due to the disordered agglomeration of BEA crystals [59]. The pore size distribution of the BEA sample, calculated from the adsorption branch using the BJH model (Figure 3b), reveals a peak at approximately 4 nm. This peak is attributed to the so-called tensile strength effect, associated with a forced closure of the hysteresis loop, evidenced by a sudden drop in the adsorbed volume at the p/p_0_ = 0.3 [61].

The modification of BEA with the NH_4_OH solution for 0.5 h resulted in a decrease in the specific surface area (S_BET_), whereas the treatment with NH_4_F or NaOH led to an increase in this parameter (Table 1). Modification with all three agents also caused an increase in mesoporosity, reflected by a higher mesopore surface area (S_ext_) and volume (V_meso_), accompanied by a loss of microporosity (V_micro_). This is evident in the pore size distribution profiles as an increased maximum at ~4 nm (Figure 3b). The most significant loss of microporosity was observed for the BEA-Na sample, where V_micro_ decreased to 0.09 cm^3^/g compared to 0.23 cm^3^/g for the initial BEA. This reduction is reflected in the nitrogen adsorption–desorption isotherms as a decrease in N_2_ uptakes at p/p_0_ < 0.1. Additionally, the BEA-Na sample exhibited a pronounced slope in the p/p_0_ range of 0.4–0.9, with a distinct H3 hysteresis loop associated with capillary condensation. Such isotherms indicate the formation of a hierarchical micro-mesoporous structure containing both micropores and mesopores. BJH analysis revealed a broad distribution of mesopores with a maximum at 40 nm (Figure 3b). These mesopores can be attributed to both intercrystalline voids between the nanocrystallites in BEA aggregates and intracrystalline pores within the crystals. In contrast, the BEA-NH and BEA-F samples exhibited only a slight increase in the hysteresis loop in the 0.3–0.8 range, suggesting the formation of less pronounced mesoporosity. The modifications with NH_4_OH and NH_4_F also led to minor changes in the hysteresis loops at p/p_0_ > 0.8, while the NaOH treatment caused significant alterations in this region, highlighting differences in the extent and nature of mesoporosity formation depending on the modifier used. The relatively low decrease in microporosity (V_micro_) and the preservation of N_2_ adsorption uptakes at low relative pressures for NH_4_OH- and NH_4_F-modified samples suggested a low decrease in their crystallinity, as confirmed by XRD analysis [52]. The observed decrease in micropore volume for all the modified samples aligned with a reduction in their crystallinity in agreement with XRD and FTIR data.

In addition to the type of modifier, the modification time also significantly influences the porous structure. For the BEA samples modified with NH_4_OH and NH_4_F solutions, the impact of modification time (0.5, 1, and 2 h) was analyzed, and the results are summarized in Table 1, Figures S1 and S2. For the BEA-NH series, the specific surface area increased with modification time, from 468 m^2^/g for BEA-NH to 545 m^2^/g for BEA-NH-2. This increase was accompanied by a notable rise in mesopore volume (V_meso_), indicating the development of additional porosity. These changes are reflected in the pore size distribution (Figure S1), where a broad peak in the range of 30–50 nm is observed. In contrast, for the samples modified with the NH_4_F solutions, the specific surface area decreased with longer modification times, from 557 m^2^/g for BEA-F to 477 m^2^/g for BEA-F-2. These changes were not accompanied by significant alterations in V_micro_ or V_meso_. Pore size distribution analysis (Figure S2b) showed that a broad peak in the range of 30–50 nm appeared only after 2 h of modification.

XRF analysis revealed, for the initial sample, a Si/Al molar ratio of 14.0. Modification with different chemical agents resulted in notable alterations in the material’s composition, as reflected in the Si/Al ratios of the modified samples (Table 1). The most pronounced change was observed for the sample treated with the NaOH solution, where the Si/Al ratio decreased to 9.6. This suggests extensive silica extraction from the zeolite framework due to alkaline desilication, leading to the highest mesopore formation among the modified samples. Additionally, a substantial drop in solution pH (from 13.30 to 11.93) after modification indicates a reaction between silica and NaOH, causing its dissolution in the alkaline environment. The treatment with NH_4_OH caused only a slight reduction in the Si/Al ratio to 13.8, and this value remained unchanged even when the modification time was extended to 1 h and 2 h. Meanwhile, the pH of the NH_4_OH solution dropped from 11.90 to 10.99, indicating some interaction between the base and the zeolite, although the silica removal was much less pronounced compared to the NaOH treatment. This suggests that NH_4_OH, being a weaker base, does not induce significant desilication like NaOH, and therefore, its modification does not substantially affect the material’s chemical composition, even after prolonged exposure. However, the observed increase in mesoporosity with treatment duration suggests a continuous removal of framework atoms, but, in addition to silica extraction, probably some aluminum is also removed. These processes occur proportionally, leading to the maintenance of a constant Si/Al ratio. Similar aluminum extraction during desilication has been reported in previous studies [16,62]. A different pattern was observed when the zeolite BEA was treated with the NH_4_F solution. In this case, the Si/Al ratio initially increased to 16.5, after 0.5 h of treatment, and then gradually decreased to 14.3 with extended treatment time. The increase in the Si/Al ratio suggests preferential removal of aluminum from the zeolite framework. This process was accompanied by a slight rise in the pH of the solution after modification (by approximately 0.1 unit, from 7.38 to 7.50), suggesting the limited extraction of framework atoms, which is further confirmed by nitrogen adsorption analysis, indicating the lowest mesoporosity compared to the samples modified with hydroxides. The gradual decline in the Si/Al ratio in samples treated for 1 h and 2 h suggests a more complex fluoride–zeolite interaction, possibly involving initial dealumination followed by partial reinsertion of Al into the framework. Similar observations have been reported by other researchers [16]. The obtained XRF results confirm that different modifying agents can be used to precisely tailor the properties of the zeolite depending on the desired chemical and structural changes in the material.

**Figure 4:**
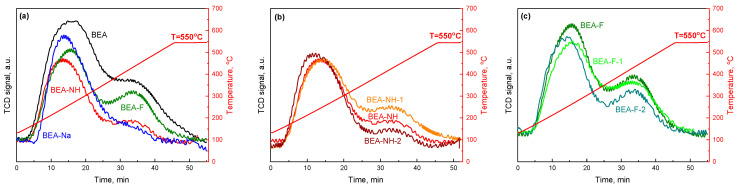
NH_3_-TPD profiles of the unmodified and modified BEA samples: modified with different modifiers for 0.5 h (**a**), modified with NH_4_OH (**b**), and modified with NH_4_F (**c**) for different time.

To investigate the acidic properties of the samples, the initial and modified Beta zeolites were characterized using NH_3_-TPD measurements. The NH_3_-TPD profiles and the results of their deconvolution are presented in Figure 4 and Figure S3, while Table 2 summarizes the corresponding amounts of desorbed NH_3_, calculated by integrating the relevant profiles, along with the density of acidic sites. The TPD profiles for all the samples exhibited a similar pattern, which can be deconvoluted into three peaks with maxima at 200–250 °C, 250–350 °C, and >350 °C, corresponding to at least three types of acid sites. According to the traditional classification [63], these peaks represent two categories of acid strength: NH_3_ desorbed below 350 °C corresponds to weak acid sites (low–temperature peaks, LT), while NH_3_ desorbed above 350 °C corresponds to strong acid sites (high–temperature peak, HT). The first deconvoluted peak (200–250 °C) is associated with the desorption of ammonia interacting with silanol groups of weak acid strength, while the second peak (250–350 °C) arises from ammonia desorbed from coordinatively unsaturated cationic species acting as Lewis acid sites. The third peak (HT), at temperatures > 350 °C, corresponds to ammonia bonded to bridging hydroxyl groups associated with framework aluminum, characteristic of Brønsted acid sites. All the modified samples exhibited lower total acidity compared to the untreated BEA. The most significant reduction in acidity, amounting to approximately half of the initial sample’s value, was observed for the sample modified with NaOH. Modification with NH_4_F resulted in a less pronounced decrease in acidity. For samples in this series, total acidity remained largely unaffected by modification time (~650 µmol/g). The reduction in acidity primarily involved a decrease in weak silanol and Brønsted acid sites, although the decrease in Brønsted acid sites was less pronounced compared to the NH_4_OH-modified samples. In the case of the NH_4_OH-modified samples, a greater reduction in total acidity was observed relative to the NH_4_F-treated series. This reduction was primarily due to a decrease in both Lewis and Brønsted acid sites, with little or no change in weak silanol acid sites, except for the sample treated for 1 h (BEA-NH-1).

**Figure 5:**
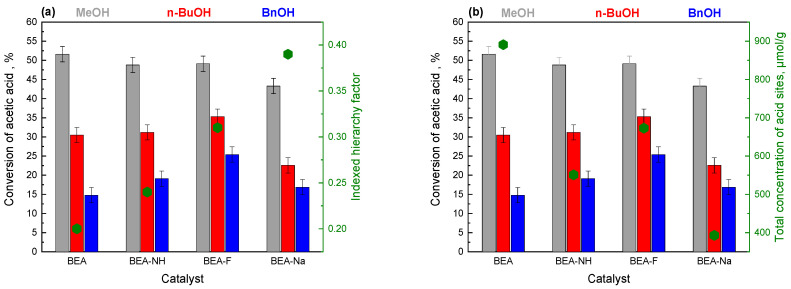
Conversion of acetic acid in the esterification reaction with different alcohols (MeOH—gray, n-BuOH—red, and BnOH—blue) on a series of BEA catalysts as a function of the indexed hierarchy factor (**a**) and the total concentration of acid sites (**b**).

Figure 5a,b present the catalytic activity of the initial and modified BEA materials in the esterification reaction of acetic acid with methanol (MeOH), n-butanol (n-BuOH), and benzyl alcohol (BnOH). For all systems, the reaction was carried out for 4 h at 70 °C for the MeOH and 90 °C for the n-BuOH and BnOH.

The conversion of the acetic acid was primarily influenced by the type of alcohol used. Methanol resulted in the highest conversions, ranging from 45–55%, regardless of the catalyst. Lower conversions, between 20–35%, were observed for n-BuOH, while the lowest conversions were noticed for BnOH, with values not exceeding 25%. This decrease in conversion is attributed to the increasing size of the alcohol molecules, which leads to greater spherical hindrance.

**Figure 6:**
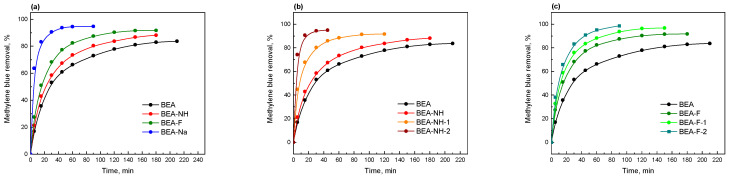
Adsorption of MB on investigated samples as a function of time: samples modified with different modifiers for 0.5 h (**a**), samples modified with NH_4_OH (**b**), and samples modified with NH_4_F (**c**) for different time.

An important parameter influencing the conversion of acetic acid was the type of modifier used for the modification of the initial BEA, which affected not only the porous structure but also the total surface acidity. In the esterification reaction with the small molecule methanol, the catalytic activity of the materials was closely related to their acidity, as all active sites were accessible to both reactants. The highest activity was observed for the unmodified BEA, while the lowest was recorded for the BEA-Na sample (Figure 5, Table 2). When n-BuOH and BnOH were used, the conversion of acetic acid was higher for the NH_4_OH-modified systems compared to the unmodified sample. This increase in activity can be attributed to the mesoporosity development, as evidenced by the rise in the indexed hierarchy factor (Figure 6a) and the improved accessibility of active sites for bigger molecules. For BEA, the factor was 0.20, while for BEA-NH, it increased to 0.24. A higher increase in the hierarchy factor was recorded for BEA-F (0.31), which resulted in an even higher conversion of acetic acid compared to BEA-NH.

Another factor influencing activity in the esterification reaction of the acetic acid was the concentration of acid sites, as illustrated in Figure 5b. The unmodified BEA exhibited the highest acidity; however, with the lowest indexed hierarchy factor, this catalyst demonstrated considerably lower activity than the modified BEA in the esterification reaction with n-butanol and benzyl alcohol. When comparing BEA-NH and BEA-F, it was found that the latter had a 20% higher concentration of acid sites, which resulted in its superior activity compared to BEA-NH in esterification with n-BuOH and BnOH. The lowest conversions in acetic acid esterification were recorded for the BEA-Na system. This catalyst exhibited the highest mesopore content (with the index of hierarchy being 30% higher than for BEA-F and almost 40% higher than for BEA-NH) but the lowest total concentration of acid sites at 393 µmol/g, compared to 551 and 673 µmol/g for BEA-NH and BEA-F, respectively.

A parameter influencing the activity of BEA catalysts in the esterification of acetic acid was the modification time. The results are presented in Figure S4a–d. For the BEA-NH series, increasing the modification time from 0.5 to 2 h led to a decrease in acidity (Figure S4a) and an increase in the hierarchy factor (Figure S4b). These changes resulted in a slight increase in the activity of the NH_4_OH-modified samples with a longer modification time in acetic acid esterification using BnOH, which can be attributed to the increase in mesopore surface area and enhanced accessibility of active sites. For the BEA-F series, extending the modification time also led to a decrease in acidity (Figure S4c) but it also caused a decrease in the hierarchy factor (Figure S4d). These decreases were minor and did not exceed 3% in the case of acidity, which may explain the minor changes in the activity of NH_4_F-modified materials with the longer modification time.

Considering the indexed hierarchy factor (Figure 5a) and the total concentration of acid sites (Figure 5b), it can be concluded that the combination of these two parameters facilitates the creation of a catalyst with enhanced activity compared to the initial microporous Beta zeolite, particularly when larger alcohol molecules such as n-BuOH and BnOH are used.

The effective removal of organic dyes from aqueous solutions is a critical aspect of wastewater treatment, underscoring the importance of adsorbent efficiency. Adsorption, a reversible process, involves the establishment of equilibrium between the liquid and solid phases. Key factors that define a good adsorbent include a high adsorption capacity and the ability to quickly reach equilibrium, which ensures the rapid removal of pollutants from water. In this context, the methylene blue (MB) adsorption over time on both initial and modified samples was examined.

In order to investigate the adsorption processes of MB dyes on the Beta samples, kinetic analysis was conducted using pseudo-first- and pseudo-second-order models. The linear regression coefficient (R^2^) and parameters of the kinetic models are presented in Table 3 and the linear regressions are displayed in Figures S5 and S6. The results reveal a poor fit with the pseudo-first-order model due to lower R^2^ values and a wider variation between the experimental (q_e,exp_) and calculated (q_e,c_) adsorption capacities. Conversely, a good fit was observed with the pseudo-second-order model, as indicated by the correlation coefficients (≥0.9997) and the close agreement between the theoretical (q_e,c_) and experimental (q_e,exp_) values. This conclusion applies to all the investigated samples. The fact that the kinetics of the MB adsorption on the investigated samples follows the pseudo-second-order model suggests that the rate-limiting step is the chemical interactions between the dye and the adsorbent [64,65]. The adsorption mechanism, in which MB is chemisorbed on the surface of the BEA samples, implies that methylene blue, as a cationic dye of phenothiazine type, is likely adsorbed via an ion exchange process, replacing protons from Brønsted acid sites on the adsorbents with the cationic dye molecules. A similar MB adsorption mechanism has been proposed previously [66,67].

Figure 6 and Figure S7 depict the time-resolved uptake of MB over the initial and modified Beta samples. According to the obtained kinetic profiles, the efficiency of Beta zeolite in adsorptive MB removal improved after hierarchization with different modifiers, attributed to increased accessibility to adsorption centers. As can be seen, for all the samples, an initial rapid adsorption rate was observed, corresponding to a large number of available adsorption sites. Over time, the adsorption rate gradually decreased and eventually stabilized, reaching saturation at equilibrium. The parent Beta material achieved maximum adsorption capacity after 210 min, whereas all the modified samples attained maximum capacity in a shorter time. Among all the samples modified during 30 min, the BEA-Na sample, exhibiting the highest hierarchy factor in this series, achieved maximum efficiency in just 90 min and indicated the highest efficiency in this series, removing 94.7% of the MB (Figure 6a). The adsorption processes for the BEA-NH and BEA-F samples followed similar time-dependent trends; however, the NH_4_F-modified sample exhibited higher efficiency.

The adsorption profiles also depended on the modification time of the samples. In both the NH_4_OH- and NH_4_F-modified series, shorter times to achieve maximum efficiency were observed for samples with increased modification time. The BEA-NH-2 sample, characterized by the highest hierarchy factor among all the modified samples, demonstrated the shortest time to reach maximum adsorption. Furthermore, the MB removal efficiency increased from 88.2% to 94.9% in the BEA-NH series and from 91.7% to 98.6% in the BEA-F series.

The NH_4_F-modified samples exhibited higher maximum adsorption values compared to the NH_4_OH-modified ones, despite their lower hierarchical structure. This phenomenon is likely due to the larger number of available adsorption sites, as the NH_4_F-modified samples exhibited higher concentrations of stronger acid sites (Brønsted acid sites) involved in ion exchange with the MB cations. The BEA-Na sample, despite having the lowest acid site content, exhibited a high maximum adsorption value (94.7%), attributed to its significant hierarchical structure (a high contribution of mesopores) and in consequence the accessibility of most acid sites. The BEA-F-2 sample, which demonstrated the highest MB adsorption efficiency, had a lower mesopore content but the highest concentration of strong acid sites among all the modified samples. These findings indicate that the adsorption efficiency of hierarchical BEA materials is governed by the combined effect of mesopore content and the number of strong acid sites, which are influenced by the type of modifier used to produce the hierarchical BEA materials. Additionally, the XRD analysis of the modified samples after methylene blue adsorption confirms that their structure remains intact (Figure S7), suggesting their potential stability even after the adsorption process.

## 3. Materials and Methods

### 3.1. Chemicals and Reagents

The chemicals and reagents used in this study were the following: tetraethylammonium hydroxide (35% TEAOH, Sigma-Aldrich, St. Louis, MO, USA), hydrochloric acid (37% HCl, Stanlab, Lublin, Poland), fumed silica (SiO_2_, Sigma-Aldrich), sodium aluminate (NaAlO_2_, Riedel-de-Haën, Seelze, Germany), sodium hydroxide (NaOH, POCh S.A., Gliwice, Poland, 98.8%—synthesis and modification), ammonium hydroxide (NH_4_OH, Stanlab, 99%), ammonium fluoride (NH_4_F, POCh, 99.9%), ammonium nitrate (NH_4_NO_3_, methanol (MeOH, Stanlab, 99.8%), n-butanol (n-BuOH, Eurochem BGD, Tarnów, Poland, ≥99.4%), benzyl alcohol (BnOH, Sigma-Aldrich, anhydrous, 99.8%), acetic acid (AcH, Carlo Erba, Emmendingen Germany, 99–100%), phenolphthalein (Sigma-Aldrich, ACS reagent), sodium hydroxide (0.1 M, analytical weight, Tarchem, Tarnowskie Góry, Poland), and methylene blue (MB, Sigma-Aldrich, ≥82%).

### 3.2. Synthesis of BEA

The process of synthesizing Beta zeolite started with the preparation of a mixture containing 14.7 mL of 35% tetraethylammonium hydroxide (TEAOH) and 2.25 mL of 3.7 M hydrochloric acid. To this mixture, 2.5 g of fumed silica (serving as the silicon source) was gradually added in portions, followed by stirring for 30 min. Next, 0.1906 g of sodium aluminate, dissolved in 2.9 mL of water, was introduced into the mixture, and stirring was continued for an additional 30 min. Crystallization was subsequently performed in an autoclave at 150 °C over 94 h. After this period, the autoclave was cooled, and the precipitate was washed, centrifuged, and dried. Finally, the organic residue was removed by calcination at 550 °C for 8 h in an air atmosphere. The resulting zeolite was designated as BEA.

### 3.3. Post-Synthesis Modification of Beta Zeolite

The modifications were performed using 0.2 M NaOH, NH_4_OH, and NH_4_F solutions. Beta zeolite (3 g) pre-activated at 350 °C for 2 h was refluxed with 100 mL of the modifier solution at 65 °C for 0.5 h, 1 h, and 2 h. Following the modification, the zeolite was thoroughly washed and centrifuged until the filtrate reached a neutral pH and was dried at 90 °C for 24 h.

### 3.4. Protonic Form Preparation

To obtain the hydrogen forms of the samples modified with NH_4_OH and NH_4_F solutions, the dry samples were calcined at 550 °C for 2 h, converting the ammonium cations into protons. Samples modified for 0.5 h were labeled as BEA-NH and BEA-F, corresponding to modifications with NH_4_OH and NH_4_F, respectively. Samples subjected to prolonged modification times were labeled as BEA-NH-x and BEA-F-x, where x represents the modification time (1 or 2 h).

The material modified with sodium hydroxide was subjected to a three-step ion exchange using 0.5 M NH_4_NO_3_ to obtain its hydrogen form. In this process, 1.5 g of the modified zeolite was mixed with 50 mL of 0.5 M ammonium nitrate solution and refluxed at 60 °C for 1 h. After each step, the zeolite was separated by centrifugation, and fresh NH_4_NO_3_ solution was added. This process was repeated two more times. The centrifuged and dried zeolite was then calcined at 550 °C for 2 h. This sample was labeled as BEA-Na.

### 3.5. Characterization

XRD measurements were conducted using powder X-ray diffraction on a Bruker AXS D8 Advance apparatus (Bruker, Billerica, MA, USA) equipped with a Cu lamp (Cu Kα1) emitting radiation with a wavelength of λ = 0.15406 nm. The analysis was conducted in the wide-angle (6–60°) range. The relative XRD crystallinity (%) of the modified samples was calculated based on the summation of the intensities of two diffraction peaks at 2θ = 7.8° and 22.4°, using untreated BEA as a standard (i.e., the crystallinity of 100%) [68].

The FTIR spectra were recorded using a Bruker Tensor 27 spectrophotometer (Bruker, Billerica, MA, USA). Measurements were performed using the transmission technique in the wavenumber range of 4000–400 cm^−1^ with a resolution of 1 cm^−1^. The sample (0.8 mg) was mixed with 200 mg of KBr and formed into pellets using a press (150 MPa).

N_2_ adsorption/desorption studies were conducted using a Quantachrome NOVA 1000 instrument (Quantachrome, Boynton Beach, FL, USA). Before the measurement, the samples were outgassed at 300 °C for 16 h. The specific surface area was determined using the BET method, whereas the external surface area and micropore volume were calculated by the t-plot method. The total volume of pores was assessed using the single point model (at p/p_0_ = 0.99). The mesopore volume was calculated as the difference between the total and micropore volumes (V_t_–V_micro_). The BJH pore size distributions were derived from the adsorption branch. The obtained textural data also enabled the determination of the indexed hierarchy factor (IHF), which defines the ratio of mesopores to micropores in the analyzed samples. It is calculated using the formula

(1) $$IHF=\frac{V_{micro}}{V_{micro.max}}\cdot \frac{V_{meso}}{V_{meso,max}}$$

where V_micro_ and V_meso_ represent the volumes of micropores and mesopores in the modified samples, respectively, V_micro,max_ denotes the maximum micropore volume in the unmodified zeolite, and V_meso,max_ indicates the maximum mesopore volume in the modified zeolite [52].

Si/Al ratio was determined by X-ray fluorescence (XRF) measurements using energy dispersive Micro X-ray Fluorescence Mini-Pal 2 spectrometer (PANalytical, Malvern, UK).

Temperature-Programmed Desorption of Ammonia (NH_3_-TPD) measurements were performed using a Micromeritics PulseChemiSorb 2705 apparatus with a thermal conductivity detector (TCD) (Micromeritics, Norcross, GA, USA). In a typical experiment, the sample (~300 mg) was activated in He (99.999%, Linde, Pullach im Isartal, Germany) for 1 h at 550 °C. The temperature ramp rate was 10 °C/min. Subsequently, the sample was cooled to 100 °C and saturated with ammonia (99.99%, Linde) for 15 min. Physically adsorbed NH_3_ was removed by purging with helium at 100 °C for 1 h. NH_3_-TPD analysis was performed in the temperature range of 100–550 °C with a ramp rate of 10 °C/min. All NH_3_-TPD profiles presented in this study were normalized to a sample mass of 1 g.

The catalytic activity of the samples was evaluated in the esterification of acetic acid (AcH) with three structurally different alcohols: methanol, n-butanol, and benzyl alcohol. Before the reaction, the catalysts were pre-activated in air at 350 °C for 2 h. Activated materials (0.05 g) were added to glass vials containing a mixture of AcH (20.0 mmol) and the respective alcohol (40.0 mmol) under vigorous stirring (400 rpm). The reactions were conducted at 95 °C for 4 h, except for the reaction with methanol, which was performed at 70 °C. The catalyst activity was determined by titrating the reaction mixtures with 0.1 M sodium hydroxide to calculate the amount of unreacted AcH. Prior to titration, the catalyst was separated from the solution using a 0.2 µm PTFE syringe filter (Lab Logistics, West Haven, CT, USA). A 0.5 mL aliquot of the filtered solution was transferred to a volumetric flask and diluted with 10 mL of water. Phenolphthalein was used as the indicator for alkalimetric titration. All catalytic tests were performed in triplicate under identical conditions. The conversion of AcH to the corresponding acetate ester (methyl acetate, n-butyl acetate, or benzyl acetate), the sole product of esterification, was calculated using the following equation:

(2) $$Conversion\;of\;AcH\left[\%\right]=\frac{moles\;of\;reacted\;AcH}{initial\;moles\;of\;AcH}\cdot 100\%$$

The moles of reacted AcH were determined as

(3) moles of reacted AcH=initial moles of AcH−moles of NaOH used for titration

The ability of Beta materials to adsorb organic compounds was investigated using methylene blue as a model dye pollutant. In a typical adsorption experiment, 20 mg of the sample was stirred with 50 mL of methylene blue dye solution (with an initial concentration of 20 mg/L) at 400 rpm for various time intervals until equilibrium was reached. The experiments were conducted in the dark at room temperature. The efficiency of methylene blue (MB) adsorption was determined using spectrophotometric measurements performed on a Jasco V-670 UV–Vis spectrophotometer (Jasco, Tokyo, Japan) at a wavelength of 664 nm. The adsorption capacity and removal efficiency of the adsorbents were calculated using the following equations:

(4) $$q_{t}=\frac{\left(C_{0}-C_{t}\right)*V}{m}$$

(5) $$Removal\left[\%\right]=\frac{C_{0}-C_{t}}{C_{0}}\cdot 100\%$$

where C_0_ [mg/L] is the initial concentration of methylene blue, C_e_ [mg/L] is the concentration of the dye after the given adsorption time (t), m [g] is the mass of adsorbent used, and V [L] is the volume of the dye solution. Equation (4) was also used to determine the q_e,exp_ values presented in Table 3 with C_t_ taken at equilibrium (C_t_ = C_e_).

The sorption kinetics of MB dye were analyzed using the pseudo-first-order (PFO) and pseudo-second-order (PSO) linear models [65,69]. The pseudo-first-order model assumes that the reaction rate is proportional to the number of ions present in the solution at a given time. In contrast, the pseudo-second-order model assumes a chemisorption process occurs between the adsorbent and the adsorbate. The kinetic parameters were determined by using linearized forms of the pseudo-first-order (Equation (5)) and pseudo-second-order (Equation (6)) models:

(6) $$\ln\left(q_{e}-q_{t}\right)=-k_{1}t+lnq_{e}$$

(7) $$\frac{t}{q_{t}}=(\frac{1}{q_{e}})t+(\frac{1}{k_{2}q_{e}^{2}})$$

where q_e_ and q_t_ represent the amount of adsorbate adsorbed at equilibrium and at any given time (t), respectively, while k_1_ and k_2_ correspond to the adsorption rate constant for the pseudo-first-order and pseudo-second-order models, respectively.

## 4. Conclusions

In this work, Beta zeolite was post-synthetically modified with different agents generating secondary porosity (NaOH, NH_4_OH, NH_4_F) under identical conditions (time, temperature, and modifier concentration) to compare the efficiency of these agents in creating mesopores. For the milder modifying agents, NH_4_OH and NH_4_F, the effect of treatment time was also investigated. The applied modifier influenced chemical composition, the mesoporosity and the acidity of the modified materials. The treatment with hydroxides (NaOH, NH_4_OH) primarily leads to the removal of silicon from the zeolite framework (desilication), resulting in a lower Si/Al molar ratio. In contrast, modification with NH_4_F causes the selective removal of aluminum (dealumination), leading to an increase in the Si/Al molar ratio. In all the modified samples, an increase in mesoporosity was observed, accompanied by a decrease in the number of acid sites. In the case of Beta zeolite modified with NaOH, the greatest increase in mesopore content was observed, though at the expense of the largest reduction in total acidity. On the other hand, the samples modified with NH_4_F exhibited the highest retention of acidity. Differences in the degree of mesoporosity and retained acidity translated into catalytic activity in the esterification of acetic acid with alcohols of varying molecular sizes. In the esterification reaction with methanol, a small molecule, catalytic activity was proportional to the acidity of the material and the highest activity showed the unmodified Beta. However, for esterification with larger alcohols (n-butanol, benzyl alcohol), catalytic activity was determined by both acidity and mesoporosity. Higher activities were exhibited by the materials modified with NH_4_OH and NH_4_F, which had lower mesoporosity but a higher number of retained active sites. The adsorption efficiency of large molecules, such as methylene blue (MB), also depended on the combination of mesoporosity and the number of acid sites. The best adsorption properties were exhibited by the materials modified with NH_4_F, which retained the highest number of Brønsted acid sites acting as adsorption centers. A longer modification time increased the hierarchical structure and enhanced the efficiency of the modified samples in MB adsorption and esterification, particularly for larger alcohols (n-BuOH, BnOH), by increasing the mesopore surface area and improving the accessibility of active sites. Furthermore, the influence of mesoporosity on the adsorption equilibrium rate was observed—the rate increased with the growth of the material’s porosity.

Based on the conducted studies, it can be concluded that the type of modifier used has a crucial impact on porosity and acidity. In the studied reaction and model molecule adsorption (MB), NH_4_F modification provides the best balance between maintaining acidity and developing mesoporosity, resulting in high catalytic activity and adsorption capacity.

## Figures and Tables

**Table 1:** Physicochemical characterization of parent and modified BEA samples.

Sample	Modification Agent	Si/Al ^a^	C_XRD_ ^b^ [%]	S_BET_ ^c^ [m^2^/g]	S_micro_ ^d^ [m^2^/g]	S_ext_ ^e^ [m^2^/g]	V_t_ ^f^ [cm^3^/g]	V_micro_ ^g^ [cm^3^/g]	V_meso_ ^h^ [cm^3^/g]	IHF ^i^
BEA	-	14.0	100	529	455	74	0.36	0.23	0.13	0.20
BEA-NH	NH_4_OH	13.8	95	468	379	89	0.38	0.19	0.19	0.24
BEA-NH-1	NH_4_OH	13.8	76	492	357	135	0.56	0.18	0.38	0.46
BEA-NH-2	NH_4_OH	13.8	67	545	346	199	0.64	0.17	0.47	0.53
BEA-F	NH_4_F	16.5	101	557	429	128	0.43	0.21	0.22	0.31
BEA-F-1	NH_4_F	15.8	94	494	402	92	0.38	0.21	0.17	0.24
BEA-F-2	NH_4_F	14.3	92	477	355	122	0.37	0.18	0.19	0.23
BEA-Na	NaOH	9.6	57	657	181	476	0.74	0.09	0.65	0.39

^a^ measured by XRF, ^b^ XRD crystallinity defined as the ratio of the intensities of the most intense reflections in the modified samples and the corresponding reflections in the unmodified sample, ^c^ BET specific surface area, ^d^ surface of micropores, ^e^ external surface, ^f^ total pore volume, ^g^ micropore volume, ^h^ mesopore volume, ^i^ indexed hierarchy factor defined as (V_mic_/V_mic,max_) × (V_mes_/V_mes,max_), where V_mic_ and V_mes_ are the micropore volume and mesopore volume, while V_mic,max_ refers to the maximum micropore volume of the untreated zeolites, and V_mes,max_ refers to the maximum mesopore volume among all modified samples.

**Table 2:** The number of acid sites evaluated by temperature-programmed desorption of NH_3_ of parent and modified BEA samples.

Sample	Concentration of Acid Sites, μmol/g			Total Concentration of Acid Sites, μmol/g	Density of Acid Sites, µmol/m^2^
	T ≤ 250 °C	250–350 °C	T ≥ 350 °C		
BEA	103	367	421	891	1.68
BEA-NH	98	349	129	551	1.23
BEA-NH-1	62	296	213	571	1.16
BEA-NH-2	100	249	177	516	0.95
BEA-F	49	360	265	673	1.21
BEA-F-1	53	337	277	667	1.35
BEA-F-2	52	326	269	647	1.37
BEA-Na	93	199	101	393	0.60

**Table 3:** Kinetic parameters for the methylene blue adsorption on the investigated Beta samples.

Sample	q_e,exp_ ^a^ mg/g	Pseudo-First-Order Model			Pseudo-Second-Order Model		
		k_1_ 1/min	q_e,c_ ^b^ mg/g	R^2^	k_2_ 1/min	q_e,c_ ^b^ mg/g	R^2^
BEA	46.14	0.0810	30.31	0.9111	0.00091	46.73	0.9998
BEA-NH	47.94	0.0115	19.08	0.9358	0.00107	48.31	0.9999
BEA-NH-1	48.28	0.0186	18.03	0.8535	0.00351	48.78	0.9999
BEA-NH-2	48.50	0.0560	10.28	0.8323	0.01441	49.26	0.9998
BEA-F	48.59	0.0143	23.46	0.8978	0.00155	49.75	0.9997
BEA-F-1	49.36	0.0253	26.94	0.9409	0.00171	52.36	0.9999
BEA-F-2	50.63	0.0526	40.12	0.9652	0.00195	54.64	0.9999
BEA-Na	47.43	0.0393	10.91	0.8544	0.00852	48.78	0.9999

^a^ maximum adsorption capacity of the sample derived from the experiments, ^b^ maximum adsorption capacity of the sample calculated from the kinetic model.

## Data Availability

The data presented in this study are available on request from the corresponding author.

## Supplementary Materials

The following are available online at https://www.mdpi.com/article/10.3390/molecules30051030/s1, Figure S1: N_2_ adsorption/desorption isotherms (a) and pore size distribution (b) for BEA and BEA-NH samples modified for different time; Figure S2: N_2_ adsorption/desorption isotherms (a) and pore size distribution (b) for BEA and BEA-F samples modified for different time; Figure S3: The NH_3_-TPD profiles and their deconvolution for BEA (a), BEA-F (b), BEA-F-1 (c), BEA-F-2 (d), BEA-NH (e), BEA-NH-1 (f), BEA-NH-2 (g), and BEA-Na (h) samples; Figure S4: Conversion of acetic acid in the esterification reaction with different alcohols (MeOH—gray, n-BuOH—red and BnOH—blue) for BEA-NH and BEA-F catalysts modified for different time as a function of the total concentration of acid sites (a,c) and indexed hierarchy factor (b,d); Figure S5: Fitting of kinetic data to the pseudo-first-order model for the adsorption of MB dye onto the specified Beta samples: modified with different modifiers for 0.5 h (**a**), modified with NH_4_OH (**b**), and modified with NH_4_F (**c**) for different time.; Figure S6: Fitting of kinetic data to the pseudo-second-order model for the adsorption of MB dye onto the specified Beta samples: modified with different modifiers for 0.5 h (**a**), modified with NH_4_OH (**b**), and modified with NH_4_F (**c**) for different time.; Figure S7: Comparison of MB adsorption on all investigated samples; Figure S8: XRD patterns of selected samples after MB adsorption.
