# Supplementary material for: Rational Design of Hierarchical Beta Zeolites via Post-Synthesis Treatments and Their Applications
Source: Molecules. 2025 Feb 24;30(5):1030. doi: 10.3390/molecules30051030 (PMC11902114; doi:10.3390/molecules30051030)
Supplement: Supplementary file 1 [file molecules-30-01030-s001.zip › molecules-3479153-supplementary materials.pdf]

Supplementary Materials

Rational Design of Hierarchical Beta Zeolites via Post-Synthesis Treatments and Their Applications

Michał Zieliński, Natalia Matysiak and Ewa Janiszewska *

Faculty of Chemistry, Adam Mickiewicz University, Uniwersytetu Poznańskiego 8, 61-614 Poznań, Poland; mardok@amu.edu.pl (M.Z.); natmat6@st.amu.edu.pl (N.M.)

* Correspondence: eszym@amu.edu.pl

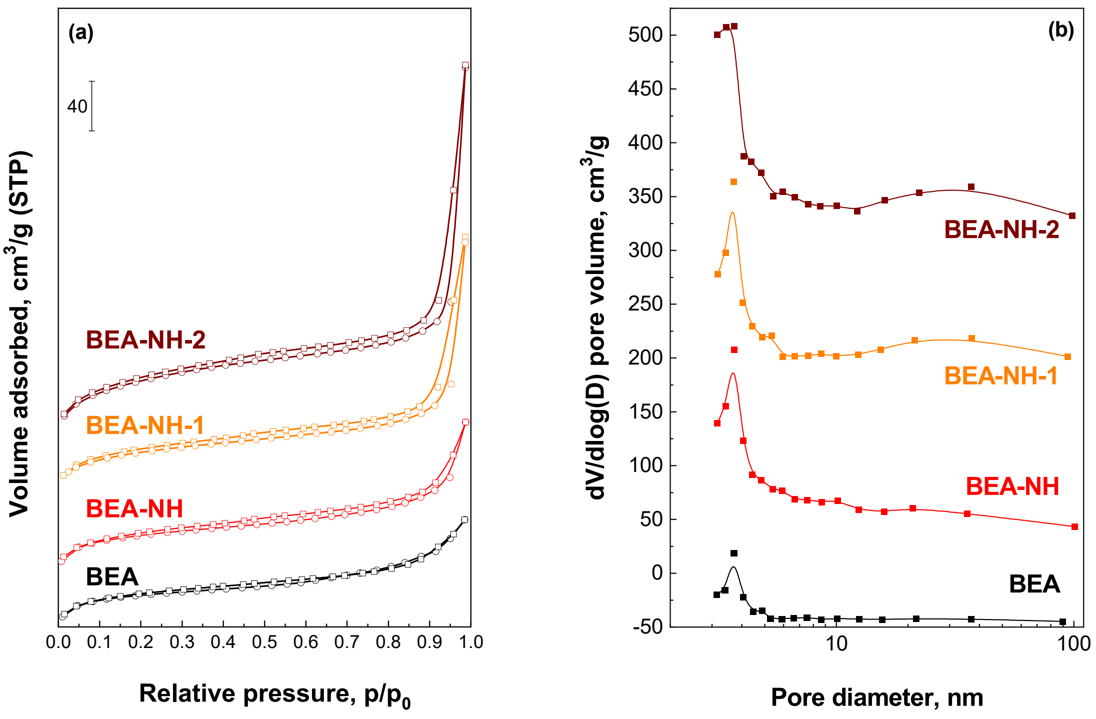

Figure S1. N_2 adsorption/desorption isotherms (a) and pore size distribution (b) for BEA and BEA-NH samples modified for different time.

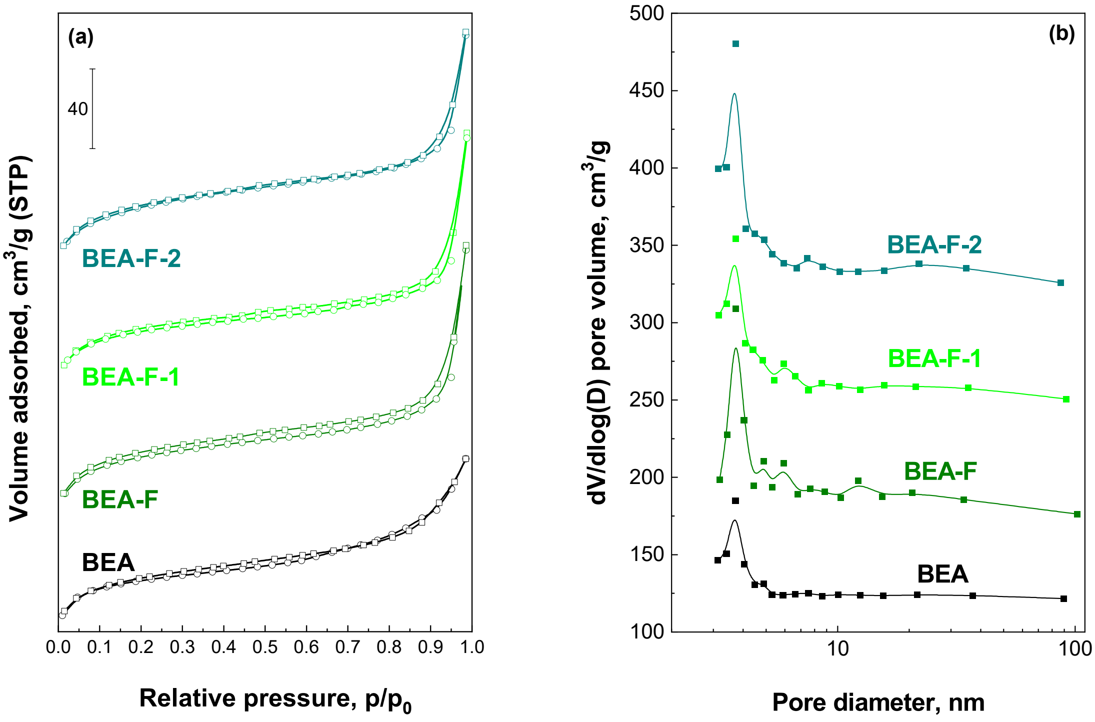

Figure S2. N_2 adsorption/desorption isotherms (a) and pore size distribution (b) for BEA and BEA-F samples modified for different time.

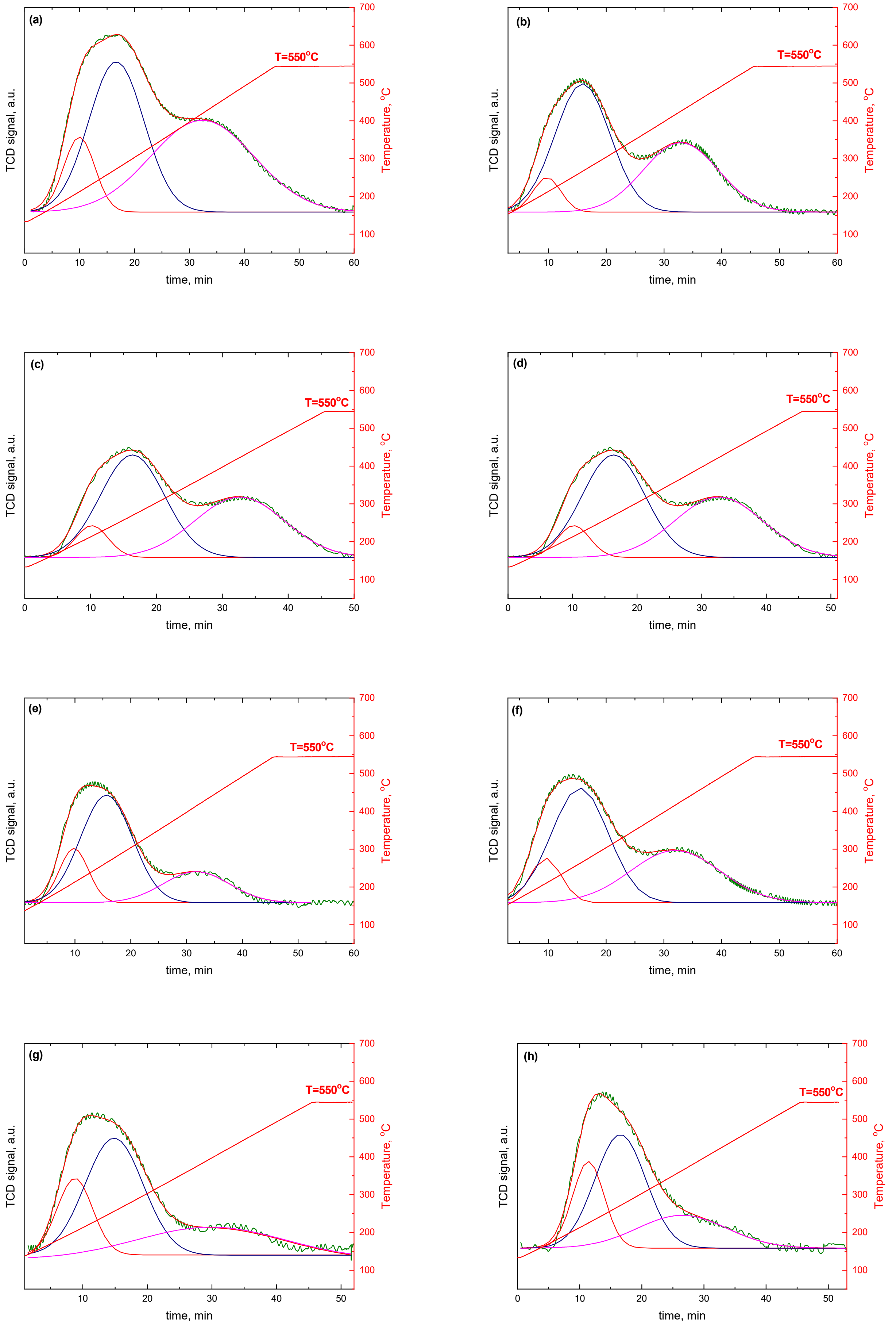

Figure S3. The NH_3-TPD profiles and their deconvolution for BEA (a), BEA-F (b), BEA-F-1 (c), BEA-F-2 (d), BEA-NH (e), BEA-NH-1 (f), BEA-NH-2 (g), BEA-Na (h) samples.

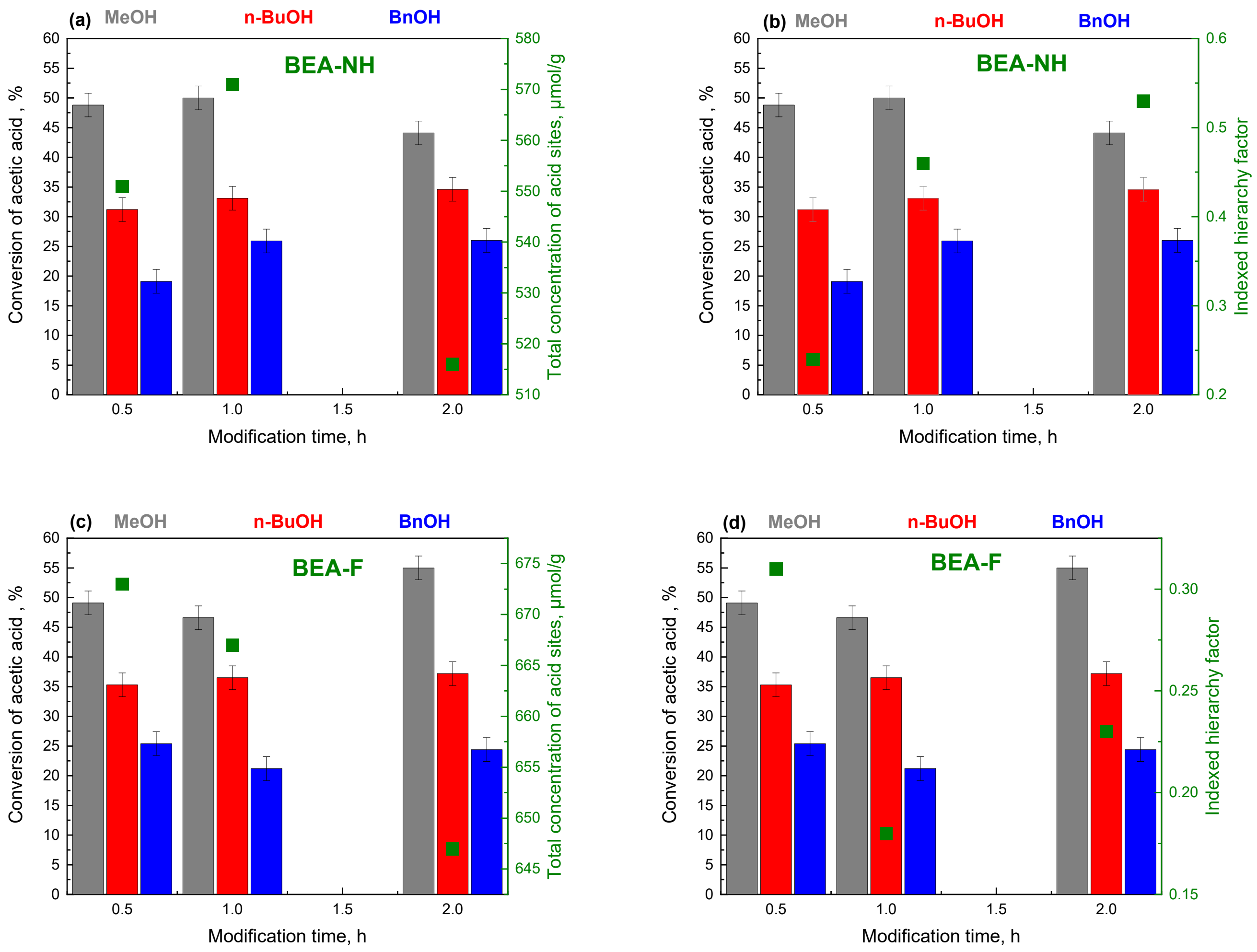

Figure S4. Conversion of acetic acid in the esterification reaction with different alcohols (MeOH - gray, n-BuOH - red and BnOH – blue) for BEA-NH and BEA-F catalysts modified for different time as a function of the total concentration of acid sites (a and c) and indexed hierarchy factor (b and d).

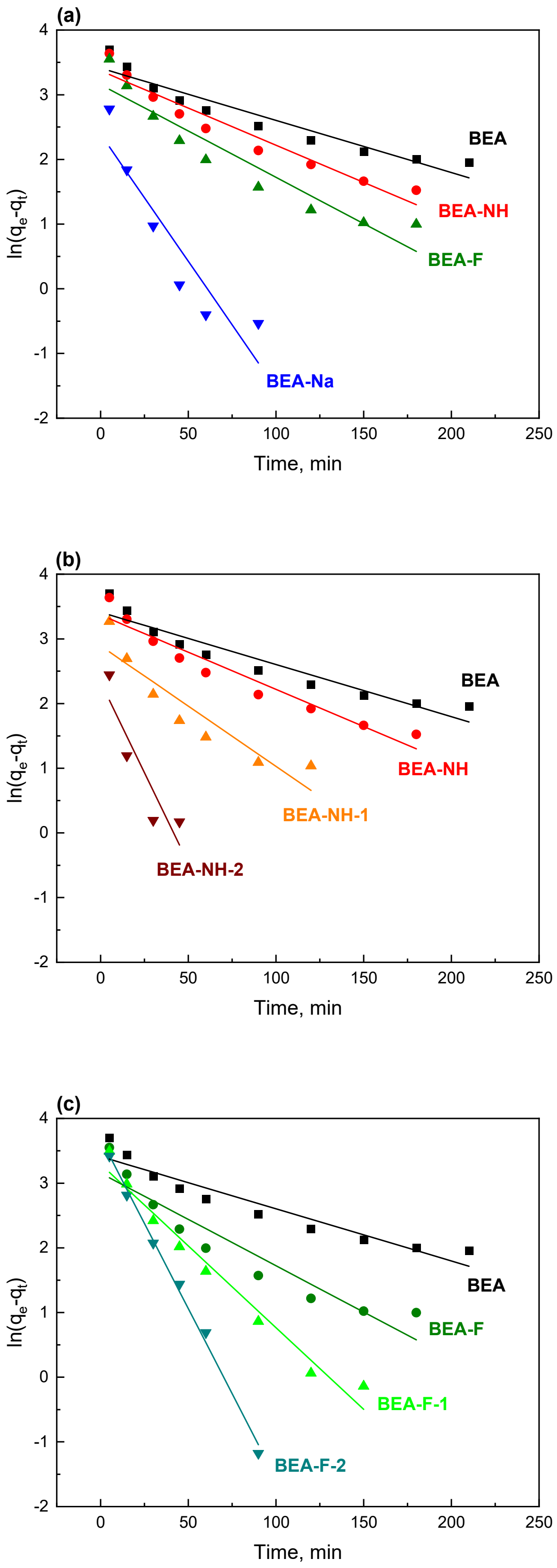

Figure S5. Fitting of kinetic data to the pseudo-first-order model for the adsorption of MB dye onto the specified Beta samples: modified with different modifiers for 0.5 h (**a**), modified with NH_4OH (**b**), and modified with NH_4F (**c**) for different time.

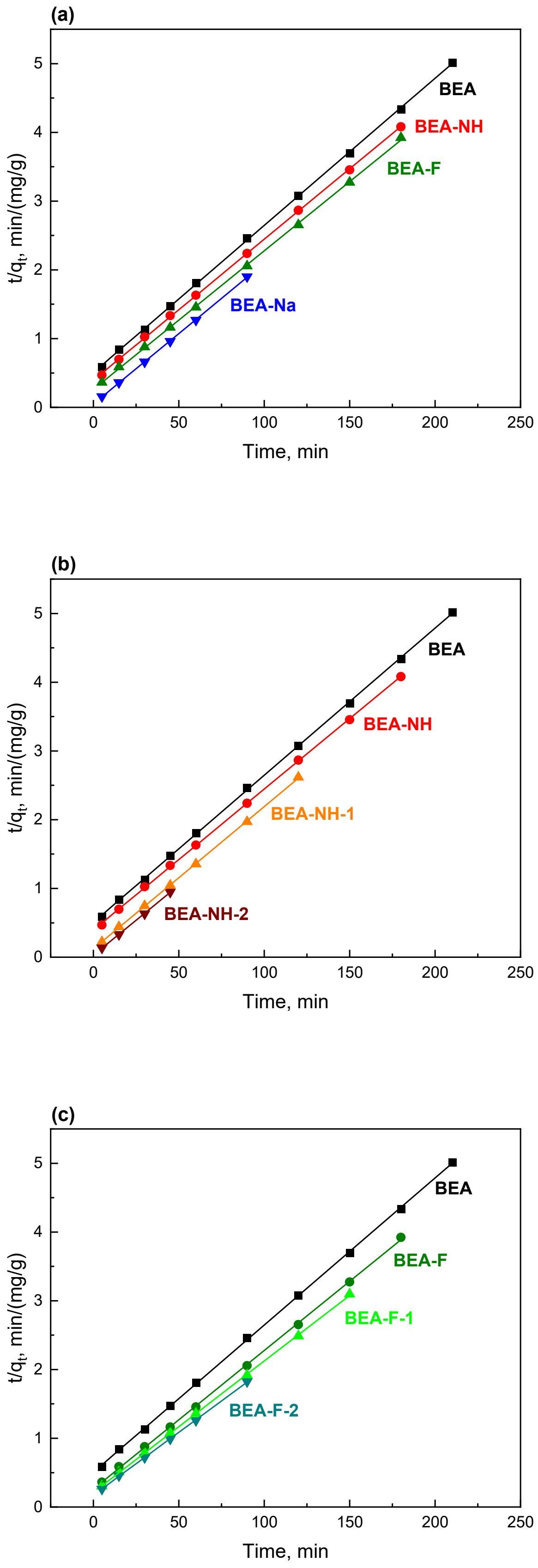

Figure S6. Fitting of kinetic data to the pseudo-second-order model for the adsorption of MB dye onto the specified Beta samples: modified with different modifiers for 0.5 h (**a**), modified with NH_4OH (**b**), and modified with NH_4F (**c**) for different time.

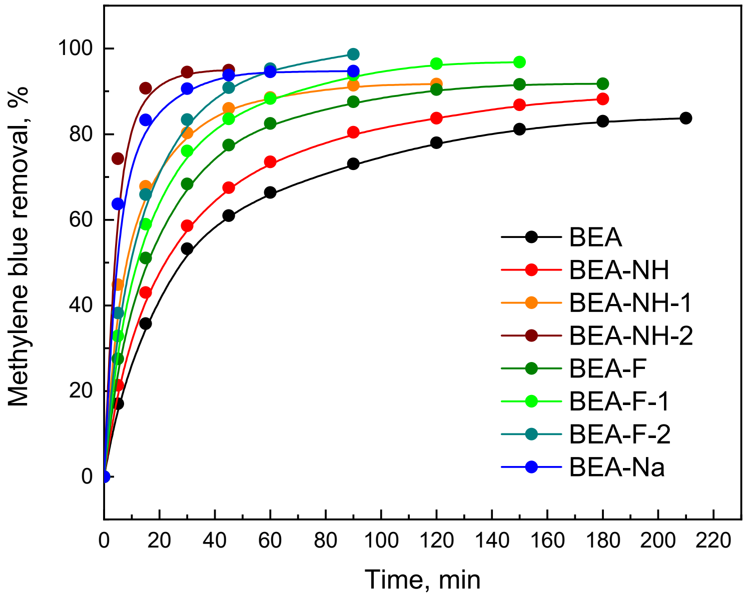

Figure S7. Comparison of MB adsorption on all investigated samples.

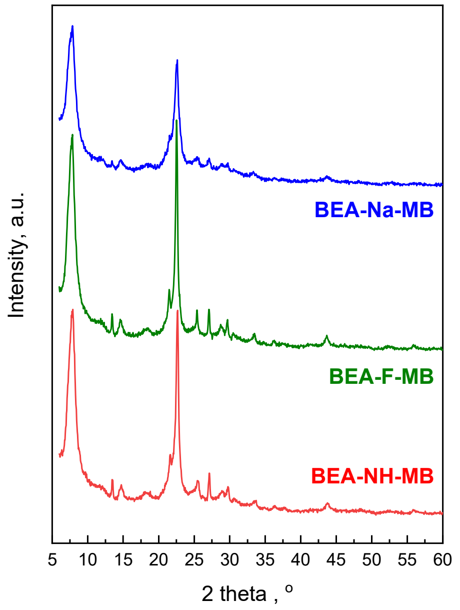

Figure S8. XRD patterns of selected samples after MB adsorption.
